# Surgical Items

**Published:** 1859-12

**Authors:** 


### SURGICAL ITEMS.

1. *Reductions of Dislocations of the Hip Joint by Manipulation.*

Since making the note published in our issue for October, a case of this accident has fallen under our notice, which differs in some respects from those heretofore met with. It was that of Page, the baggage-man, injured at the accident on the Chicago and Northwestern R. R., near Watertown, Wis. The head of the bone lay in the ischiatic notch. Near three days had elapsed since the accident. Efforts at reduction had been made by a surgeon from Milwaukee, who, after applying, as we were informed, a very great mechanical power, without success, pronounced it a *fracture of the neck of the femur*, but left it without applying any dressing. The ligaments of the hip joint appeared to be entirely torn across and much contusion produced, as the member could be moved more freely than is usual in such cases.

Reduction by Reid's method was effected, but less readily than is usual. After the reduction, a slight tetanic spasm supervened, which lasted several days, but which, at our last visit, Nov. 24, three weeks after the accident, had almost entirely disappeared.*

* We have felt called upon in this case, contrary to uniform practice, to

2. *Exsection of five Tarsal Bones for Caries.*

J. B., aged 26 years, was received into the Chicago City Hospital, Nov. 8, 1859, on account of a chronic enlargement of the bones of the tarsus of the right foot. The disease was of eighteen months' standing, and involved the bones between the head of the metatarsus and the ankle. The patient was in good health ; had suffered little pain.

Wednesday, Nov. 9, 1859, the operation was performed as follows : An incision was made across the tarsus at its junction with the metatarsus, and a flap raised toward the ankle. The three cuneiform, the naviculare and the cuboid bones were found diseased, and removed. Suppuration had already taken place between the middle cuneiform and the second metatarsal bone, the head of which was cut away with the cutting forceps. The *arteria dorsalis pedis* alone required a ligature. The edges of the wound were stitched together, and the member placed in a fracture box. No accident occurred. The wound is at the present time nearly cicatrized, and the foot only appears shortened.

3. *Strangulated Femoral Hernia.*

This was an old hernia in a patient, 62 years of age. A truss had been worn without the reduction being fully effected, so that a mass of omentum had become intimately adherent to the sac, so much so that this was hardly distinguishable. One mass of omentum passed upward and outward above Poupart's ligament, another in the opposite direction, while in the midst was a small knuckle of intestine of recent protrusion.

The strangulation had existed three days, and frequent efforts at reduction had been made. The separation and reduction of the omentum after the stricture was divided, was a work of much difficulty, but this was at length effected. The operation was performed, Nov. 19, 1859. Up to the present date (Nov. 22) the patient is doing well.

---

make a statement of facts, which may seem to reflect upon the surgeon previously called, in consequence of the attacks repeatedly made upon us in the *Milwaukee Daily News.*

4. *Amputation at the Hip Joint.*

This, the third we have had occasion to do, was performed Nov. 16, at the City Hospital. The patient, a woman, aged 30 years, had a large encephaloid tumor of the left lower member, which extended from the middle of the leg to near the hip. A bony and brain-like tumor, of large size, had been removed from behind the knee of this patient eighteen months previously, by Prof. Freer, and she remained well for about one year, when the commencement of the present growth was perceived.

The vascularity of the tissues, the vessels of which were of immense size, rendered the operation difficult, and it was necessary to tie the principal arteries before finishing the operation, which was completed, however, without unusual loss of blood, owing to the care of Professors Freer and Rea, in compressing the vessels as soon as divided.

For forty-eight hours the patient did well, but at the end of that time tympanitis and nausea with vomiting occurred, and she died sixty hours after the operation.

5. *Resection of the Head of the Humerus for Gunshot Wounds.*

November 27, we were called to consult about the case of W. B., of Kendall county, Illinois, who, thirty-two hours previously, had received a charge of buckshot in the left shoulder, immediately below the head of the *humerus*. The charge was supposed to have been fired with the intention of committing murder. The bone was extensively shattered, some of the shot glanced, and came out in front on the left side of the chest. Some appeared to have penetrated the thorax, as there was emphysema, and had been some expectoration of blood. As the sensibility and temperature of the member were preserved, we advised the removal of the shattered fragments, and performed the operation. As the fracture extended to the head of the bone, this was also removed.

---

☞ The article by Dr. Gibbs on the use of white lead in burns should have appeared under the head of selections instead of that of original articles;—it had already been published in that excellent journal the *N. Y. Med. Monthly*.

We have recently used it in the City Hospital on a case which seemed quite hopeless from its extent, and it seemed for several days to exert a very favorable influence on the condition of the patient, although his condition is still doubtful.

